# Long-Term Efficacy and Impact on Mortality of Remote Magnetic Navigation Guided Catheter Ablation of Ventricular Arrhythmias

**DOI:** 10.3390/jcm10204695

**Published:** 2021-10-13

**Authors:** Denise Guckel, Sarah Niemann, Marc Ditzhaus, Stephan Molatta, Leonard Bergau, Thomas Fink, Vanessa Sciacca, Mustapha El Hamriti, Guram Imnadze, Philipp Steinhauer, Martin Braun, Moneeb Khalaph, Georg Nölker, Philipp Sommer, Christian Sohns

**Affiliations:** 1 Clinic for Electrophysiology, Herz- und Diabeteszentrum NRW, Ruhr-Universität Bochum, 32545 Bad Oeynhausen, Germany; dguckel@hdz-nrw.de (D.G.); sarah.niemann-s6d@rub.de (S.N.); smolatta@hdz-nrw.de (S.M.); lbergau@hdz-nrw.de (L.B.); tfink@hdz-nrw.de (T.F.); vsciacca@hdz-nrw.de (V.S.); melhamriti@hdz-nrw.de (M.E.H.); gimnadze@hdz-nrw.de (G.I.); mbraun@hdz-nrw.de (M.B.); mkhalaph@hdz-nrw.de (M.K.); g.noelker@hospitalverbund.de (G.N.); psommer@hdz-nrw.de (P.S.); 2 Fakultät Statistik, Technische Universität Dortmund, 44227 Dortmund, Germany; marc.ditzhaus@tu-dortmund.de (M.D.); philipp.steinhauer@tu-dortmund.de (P.S.); 3 Clinic for Internal Medicine II/Cardiology, Christliches Klinikum Unna Mitte, 59423 Unna, Germany

**Keywords:** ventricular tachycardia, remote magnetic navigation, Stereotaxis, VT ablation, heart failure, ischemic cardiomyopathy, non-ischemic cardiomyopathy, dilated cardiomyopathy

## Abstract

Remote magnetic navigation (RMN) facilitates ventricular arrhythmia (VA) ablation. This study aimed to evaluate the long-term efficacy of RMN-guided ablation for ventricular tachycardia (VT) and premature ventricular contractions (PVC). A total of 176 consecutive patients (mean age 53.23 ± 17.55 years, 37% female) underwent VA ablation for PVC (132 patients, 75%) or VT (44 patients, 25%). The cohort consisted of 119 patients (68%) with idiopathic VA, 31 (18%) with ischemic (ICM), and 26 (15%) with dilated cardiomyopathy (DCM). VA recurrence was observed in 69 patients (39%, mean age 51.71 ± 19.91 years, 23% female) during a follow-up period of 5.48 years (first quartile 770.50 days, second quartile 1101.50 days, third quartile 1615.50 days). Left ventricular ejection fraction <40% lead to a significantly increased risk for VA (*p* = 0.031*). Multivariate analyses found DCM to be an independent predictor (IP) for VA recurrence (*p* < 0.001*, hazard ratio (HR) 3.74, confidence interval (CI) 1.58–8.88). ICM resulted in a lower increase in VA recurrence (*p* = 0.221, HR 1.49, CI 0.79–2.81). Class I/III/IV antiarrhythmic drug therapy (AADs) was also identified as IP for recurrence (*p* = 0.030*, HR 2.48, CI 1.11–5.68). A total of 16 patients (9%) died within the observational period. RMN-guided ablation of VA lead to acceptable long-term results. An impaired LV function, DCM, and AADs were associated with a significant risk for VA recurrence. Personalized paths are needed to improve efficacy and outcome.

## 1. Introduction

Ventricular arrhythmias (VA) promote cardiovascular morbidity and mortality [1]. Premature ventricular contractions (PVC) and recurrent ventricular tachycardia (VT) are common in patients with ischemic and non-ischemic cardiomyopathies leading to implantable cardioverter defibrillator (ICD) therapies and worsened heart failure.

Early phases of dilated cardiomyopathy (DCM) can already be associated with life-threatening VAs [2] as well as scarred tissue caused by ischemia in patients suffering from ischemic cardiomyopathy (ICM). An appropriate ICD therapy occurs in the first year in > 50% of patients with secondary and approximately 5% of patients with primary prevention ICDs [1]. Within the last years, more and more evidence has accumulated demonstrating that structural, electrical, electromechanical, and autonomic remodeling causes complex individual arrhythmia substrates. In ICM, the probability of success of VA ablation is significantly greater (56–77%) than for patients with DCM (38–67%). Patients with idiopathic VAs show the best outcome with a success rate of more than 80% [1].

As ablation outcome depends on the exact identification and accessibility of the substrate, as well as the natural course of the disease, the recent ESC guideline suggests a pre-interventional assessment of potential arrhythmia substrates in the majority of patients [3].

In this context, cardiac magnetic resonance imaging with late gadolinium enhancement (LGE CMR) can help to identify and subsequently categorize structural changes inside the ventricular walls [1,3]. With reference to ablation techniques, VA ablation procedures are often complex and time-consuming, requiring ergonomically unfavorable positions, leading to operator fatigue [4]. Furthermore, it is often challenging to ensure permanent tissue contact during ablation. To overcome these constraints, technical advancements, including remote magnetic navigation (RMN), have been developed [5]. RMN can improve the efficacy of radiofrequency energy delivery and might reduce complication rates. This study evaluates the outcome and predictors for the success of RMN-based VA ablation procedures in a cohort of patients with idiopathic and cardiomyopathy-associated PVC and VT.

## 2. Methods

### 2.1. Patients

Consecutive patients undergoing catheter ablation guided by RMN for symptomatic VA between 2012 and 2018 were included in this retrospective study, which was performed in compliance with the principles outlined in the Declaration of Helsinki and approved by the Institutional Ethics Committee (Reg. No. 2019-458).

### 2.2. Magnetic Navigation System

The Niobe^TM^ magnetic navigation system (MNS) is a medical system manufactured by Stereotaxis, Inc. (St. Louis, MO, USA) for electrophysiological procedures in the heart. RMN facilitates the control of the distal tip of appropriately labeled compatible magnetic devices via magnetic fields, which are generated by two large neodymium-iron-boron magnets that are mounted on mechanical positioners near the head of the patient. The physician uses the MNS to steer the distal tip of the catheter, while the Cardiodrive^TM^ system provides the means for automated remote advancement or retraction. The MNS is controlled by the Navigant^TM^ Navigation Work Station software. The system is fully integrated with both fluoroscopy and electroanatomic mapping.

### 2.3. Periprocedural Management

Whenever there were no contraindications for this, patients were examined by LGE CMR prior to the procedure to evaluate the individual anatomical characteristics of the ventricles. AADs, except for amiodarone, were discontinued at least three half-lives before ablation. Anticoagulation with phenprocoumon was continued aiming for an international normalized ratio (INR) between 2.0 and 3.0. Direct oral anticoagulants (DOAC) were stopped one half-life before ablation. Pericardial effusion was ruled out immediately after ablation and 4 h later. Anticoagulation was continued within 4 h after the procedure with phenprocoumon or DOAC. AADs were prescribed to the operators’ discretion following ablation. Patients stayed in the hospital under continuous rhythm monitoring for at least 36 h.

### 2.4. Ablation Procedure

The procedure was performed under conscious sedation with propofol and analgesia with fentanyl as required. The RMN system was used to manipulate the mapping catheter and the ablation catheter from the control room.

After femoral access, a quadripolar catheter (Navistar RMT Thermocool, Biosense Webster, Inc., Diamond Bar, CA, USA) was introduced and positioned in the ventricle, and a ten-pole catheter was positioned in the coronary sinus (Inquiry, St Jude Medical, Sylmar, CA, USA).

Either the electroanatomic CARTO 3 mapping system (Biosense Webster, Inc., CA, USA) or the EnSite™ Cardiac Mapping System (St Jude Medical, Sylmar, CA, USA) was used to map the ventricle using a retrograde approach with the ablation catheter (Navistar RMT Thermocool, Biosense Webster or Trignum G, Biotronik, Berlin, Germany). The catheter was selected regardless of whether, e.g., idiopathic VA/ DCM/ ICM or VT/PVC were present.

For PVC ablation, a combination of activation mapping (detection of the earliest activation and QS complexes on unipolar map) and pacemapping was conducted. In the absence of any PVC at the beginning of the procedure, isoproterenol was administered. For pace mapping, minimal output with power (2 to a maximum of 20 mA) and minimal pulse width (0.5–2 ms) was used. If the origin of PVC was identified, ablation was performed using irrigated RFC. Following successful ablation and persistent PVC-suppression over an observational period of 30 min, programmed electrical stimulation was performed from the right ventricular apex and outflow tract to proof for non-inducibility of coexisting sustained VT. Additional VT ablation was performed in these patients when sustained VT was inducible following the ablation approach as mentioned above.

For scar-related VT, a substrate-based ablation strategy targeting local abnormal ventricular activity (LAVA) and late potentials (LP) was performed with a 3.5 mm irrigated-tip radiofrequency mapping and ablation catheter (NavistarVR RMT Thermocool, Biosense Webster or Trignum G, Biotronik, Berlin, Germany) in conjunction with the RMN system (NiobeTM, Stereotaxis, Inc., St. Louis, MO, USA). The procedural endpoint was the elimination of all LAVA judged by the operator. Acute procedural success was defined as the non-inducibility of all VA by programmed ventricular stimulation at the end of the procedure. “Partial success” was used to describe the fact that two clinically relevant VA morphologies were detected, but just one of them could be treated successfully.

### 2.5. Follow-Up

After discharge, patients underwent routine follow-up care, including ECGs, device interrogations, and interviews every 6–12 months. Unscheduled visits were conducted if required. Patients were monitored for 5.48 years (2000.00 days; 1st quartile 770.50 days, 2nd quartile 1101.50 days, 3rd quartile 1615.5 days).

### 2.6. Endpoint

We aimed to analyze the outcome of patients undergoing VA ablation procedures using RMN. The VT group included patients with recurrent symptomatic sustained (> 30 s) VT. The PVC group included patients with symptomatic non-sustained VT (< 30 s) or symptomatic PVC refractory to AAD therapy. We compared patients’ outcomes with regards to PVC compared to VT and idiopathic VT in contrast to cardiomyopathies such as DCM and ICM.

VA recurrence (defined as the recurrence of non-sustained or sustained VT as well as deterioration of PVC burden) and PVC burden was judged on (24 h)-ECG documentation, device interrogations, and symptoms suggestive for arrhythmia recurrence. Furthermore, we intended to ascertain independent predictors (IPs) of VA recurrence in this patient cohort allowing for conclusions in terms of the RMN system as well as personalized paths in VA management for certain patients.

### 2.7. Data Collection

Data on patients’ characteristics, medication, symptoms, and complications were compiled from patients’ records and discharge letters. Procedural parameters and clinical aspects concerning RMN ablation were taken from ablation protocols and procedure-related documents.

### 2.8. Statistical Analysis

All statistical analyses were performed in R version 4.0.3 with packages forestmodel, ggplot2, survival, and survminer. Median follow-up time was calculated using the reverse Kaplan–Meier approach. Continuous variables between the groups (PVC, VT, idiopathic VA, DCM, ICM) were compared by employing an unpaired two-sided Student’s t-test or Mann–Whitney test. Differences in continuous parameters between baseline and FU were analyzed by paired Student’s t-test or Wilcoxon signed-rank test. Categorical and ordinal data were examined by chi-square and Mann–Whitney tests, respectively.

Event-free survival was calculated by Kaplan–Meier analysis as time from initial RMN ablation to first documented VA recurrence during follow-up. A log-rank test was used to assess differences in event-free survival time between groups. Cox proportional hazard regression model was applied to identify IPs of arrhythmia recurrence. Demographic and clinical data from baseline analyses were included in univariate Cox proportional hazard regression models for the primary endpoint. Variables with an unadjusted association with VA recurrence (*p* < 0.1) were analyzed by multivariate Cox regression analysis. No imputation was carried out in the analysis. Data are presented as mean ± SD or percentage value unless stated otherwise. A *p*-value ≤ 0.05 was considered statistically significant.

## 3. Results

### 3.1. Patients’ Baseline Characteristics

The study population consists of 176 consecutive patients (mean age 53.23 ± 17.55 years, 37% female) undergoing RMN ablation for VA (see Table 1). A total of 132 patients (75%) suffered from symptomatic PVC (mean age 53.30 ± 15.97 years, 47% female), 44 patients (25%) (mean age 52.49 ± 21.81 years, 7% female) from recurrent VT.

119 patients (68%) presented with idiopathic VA (mean age 47.02 ± 18.19 years, 51% female) and 57 patients (32%) with cardiomyopathies (mean age 63.33 ± 10.40 years, 13% female). Among them 31 patients (18%) (mean age 65.39 ± 9.00 years, 3% female) were diagnosed with ICM and 26 patients (15%) (mean age 62.54 ± 8.70 years, 15% female) with DCM. A total of 50 patients (28%) suffered from heart failure with a reduced ejection fraction (HFrEF). Preprocedural LGE CMR was performed in 70 patients (40%).

### 3.2. Baseline Characteristics (PVC vs. VT)

Patients undergoing VT ablations presented with a significantly lower left ventricular ejection fraction (LVEF) (40.64% ± 11.84%) compared to PVC patients (50.65% ± 8.28%) (*p* < 0.001*). Far more patients of the VT group were diagnosed with ICM (47%) (*p* < 0.001*) or DCM (19%) (*p* = 0.076), had an implanted cardioverter defibrillator (ICD) (32%) or a cardiac resynchronization therapy device (CRT-D; 34%; *p* < 0.001* each) and a baseline AAD class I/III/IV therapy (71%; *p* < 0.001*). For further details on patients’ baseline characteristics see Supplementary Table S1.

### 3.3. Baseline Characteristics (Idiopathic VA vs. Cardiomyopathies (DCM, ICM))

In contrast to patients with cardiomyopathies (ICM, DCM) (mean age 63.33 ± 10.40 years) patients with idiopathic VA were significantly younger (mean age 47.02 ± 18.19 years; *p* < 0.001*), had significantly lower BMI (*p* = 0.021*) and creatinine values (*p* < 0.001*). Baseline beta blocker (90%) and AAD class I/III/IV (54%) intake was significantly higher in patients diagnosed with cardiomyopathies (DCM, ICM) (*p* < 0.001*). Patients’ baseline characteristics are summarized in Supplementary Table S2.

### 3.4. Baseline Characteristics (DCM vs. ICM)

ICM patients presented with a significantly lower renal function (*p* = 0.020*) and a significantly higher AAD class I/III/IV intake (77%, *p* = 0.015*). For further details on patients’ baseline characteristics, see Supplementary Table S3.

### 3.5. Baseline Symptoms

See Supplementary Table S4.

### 3.6. Procedural Data and Acute Procedural Success

Mean procedural duration was 206 ± 88 min, mean ablation time accounted for 19 ± 24 min with a mean radiation dose of 869 ± 2103 µGym^2^ and a mean fluoroscopy time of 5 ± 6 min.

Acute procedural success with substrate modification was achieved in 144 patients (82%). In 20 patients (11%), RMN was conducted with partial success, which means that two clinically relevant VA morphologies were detected, but just one of them could be treated successfully. In 12 patients (7%), ablation procedures were primarily not successful.

Acute procedural success did not differ significantly between the NavistarVR RMT Thermocool catheter, Biosense Webster (69 patients (83%)) and the Trignum G catheter, Biotronik (75 patients (81%)) (*p* = 0.08).

### 3.7. Impact of RMN Ablation on PVC Burden

In all patients, PVC burden was determined before and after RMN ablation. PVC burden prior ablation averaged 19%/24 h. RMN ablation led to a significant −63% reduction in PVC burden (*p* < 0.001*). No significant group differences were observed between patients with idiopathic VAs (−68%, *p* < 0.001*) and cardiomyopathies (−63%, *p* < 0.001*). Compared to DCM patients ICM patients presented with a far more distinct reduction in PVC burden (ICM: −68%, *p* = 0.002*; DCM: −37%, *p* = 0.008*). In contrast to patients with VA recurrence, patients who did not develop VA recurrence showed a significantly higher reduction in PVC (−42% vs. −84%; *p* < 0.001*).

### 3.8. Long-Term Clinical Outcome

VA recurrence occurred in 69 patients (39%; mean age 51.71 ± 19.91 years, 23% female) within the follow-up period of 5.48 years. Of them, 44 patients (64%) were primarily treated for symptomatic PVC and 25 patients (36%) for VT. The presence of cardiomyopathies (50%, *p* = 0.044*), male sex (77%, *p* = 0.039*), an impaired LVEF function <40% (39%, *p* = 0.031*), a history of stroke (10%, *p* = 0.044*), an indication for CRT-D supply (23%, *p* = 0.015*), and baseline AAD class I/III/IV intake (44%, *p* = 0.004*) were associated with significantly higher recurrence rates. The catheter used had no effect on the VA recurrence rate [Trignum G catheter, Biotronik: 39 patients (42%) vs. NavistarVR RMT Thermocool catheter, Biosense Webster: 30 patients (36%) (*p* = 0.06)].

VT patients showed a significantly higher recurrence rate (58%) compared with PVC patients (33%) (*p* = 0.007*). In the case of recurrent VA, 30 patients (43%) presented with VT, 39 patients (57%) with PVC. A total of 29 patients (42%) were scheduled for re-ablation due to symptomatic VA recurrence. A total of 10 of these patients (34%) were treated for recurrent sustained VT and 19 of them (66%) for recurrent PVC or non-sustained VT. A total of 16 patients (9%) died within the observation period. In 12 patients (75%), the exact cause of death is unclear. These patients died in the home environment; no autopsy was performed. One patient died as a result of a VT storm in the context of fulminant sepsis, another patient due to electromechanical uncoupling in progressive heart failure, another patient as a result of aspergillosis after heart transplantation.

**Figure 1:**
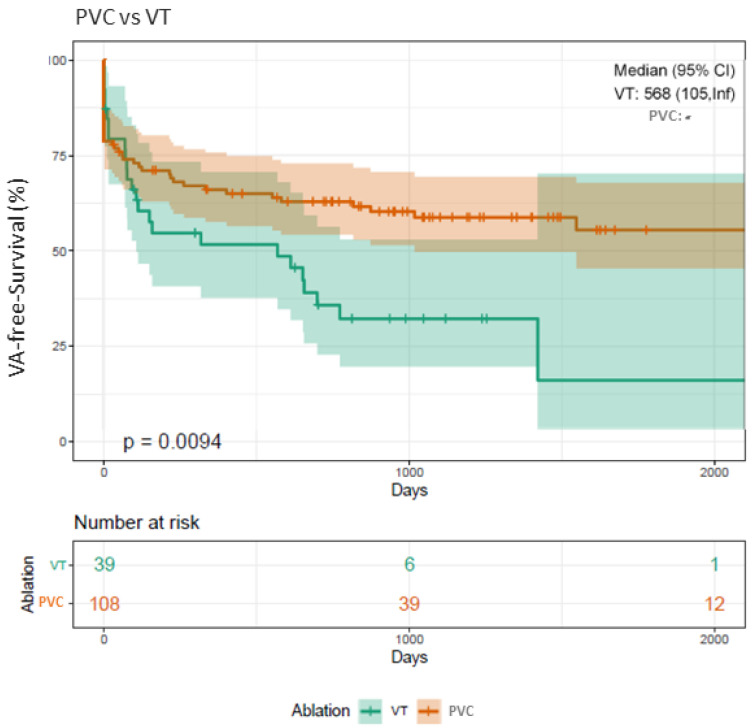
Kaplan–Meier plot on freedom from VA recurrence in patients undergoing RMN VT ablation compared to PVC ablation. Patients undergoing RMN ablation for VT present with a significantly higher recurrence rate of VA in the FU in comparison to patients treated for PVC (Log-rank *p*-value = 0.009*). VA, ventricular arrhythmia; VT, ventricular tachycardia; PVC, premature ventricular contractions; RMN, remote magnetic navigation; FU, follow-up.

The majority (12 patients, 75%) was primarily treated for VT. Kaplan–Meier analyses confirmed that patients undergoing VT ablations presented with a significantly higher risk for arrhythmia recurrence (Log-rank *p* = 0.009*) compared to patients treated for PVC (Figure 1).

### 3.9. Clinical Outcome Depending on PVC Origin

A total of 33% of PVC patients (44 of 132 patients) developed PVC recurrence. Of these, 3 patients were primarily treated for PVC from the left ventricular outflow tract (LVOT) (7%), 23 for PVC from the right ventricular outflow tract (RVOT) (52%), 10 for PVC from the left ventricle (LV) (23%), 2 for PVC from the right ventricle (RV) (5%), and 6 patients for more than one PVC morphology (14%) (5 of 6 (83%) involving the RVOT).

Thus, the frequency of recurrence depending on the site of PVC origin in decreasing order is as follows: LV (38%), RVOT (36%), RV (25%), LVOT (18%).

### 3.10. Clinical Outcome Depending on Cardiomyopathies (DCM, ICM)

**Figure 2:**
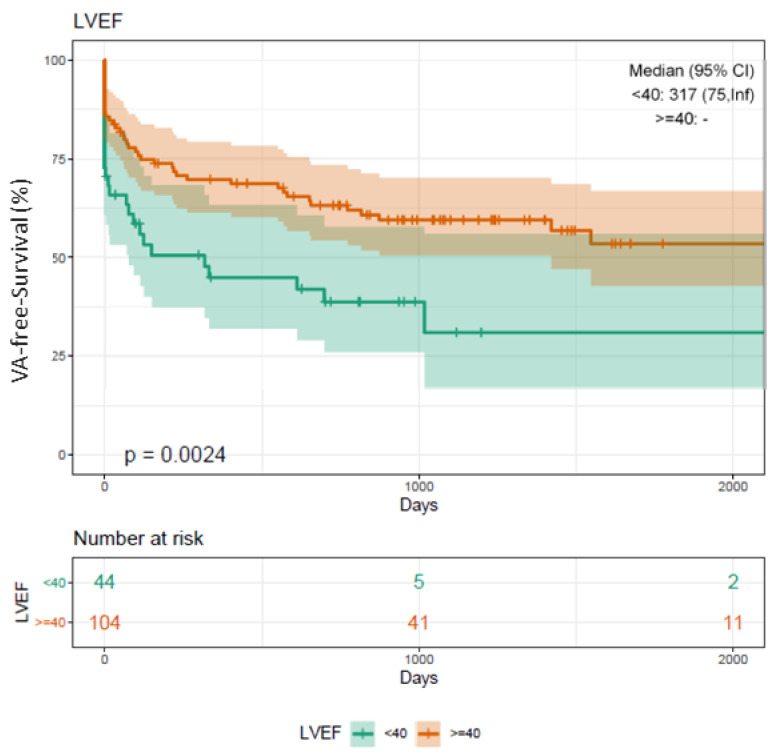
Kaplan–Meier plot on freedom from VA recurrence in patients undergoing RMN ablation depending on LVEF. Patients undergoing RMN ablation for VA with a highly reduced LVEF below 40% present with a significantly worse outcome compared to patients with an LVEF function accounting for more than 40% (Log-rank *p*-value = 0.002*). VA, ventricular arrhythmia; VT, ventricular tachycardia; PVC, premature ventricular contractions; RMN, remote magnetic navigation; LVEF, left ventricular ejection fraction; FU, follow-up.

Kaplan–Meier analyses showed that HFrEF patients are at a significantly higher risk for VA recurrence (log-rank *p* = 0.002*) compared to patients with an LVEF function >40% (Figure 2).

A total of 34 of the 69 patients with VA recurrence (49%) were diagnosed with cardiomyopathies (DCM, ICM). A total of 21 patients (30%) suffered from DCM and 13 patients (19%) from ICM. A total of 35 patients (51%) presented with recurrent idiopathic VA.

**Figure 3:**
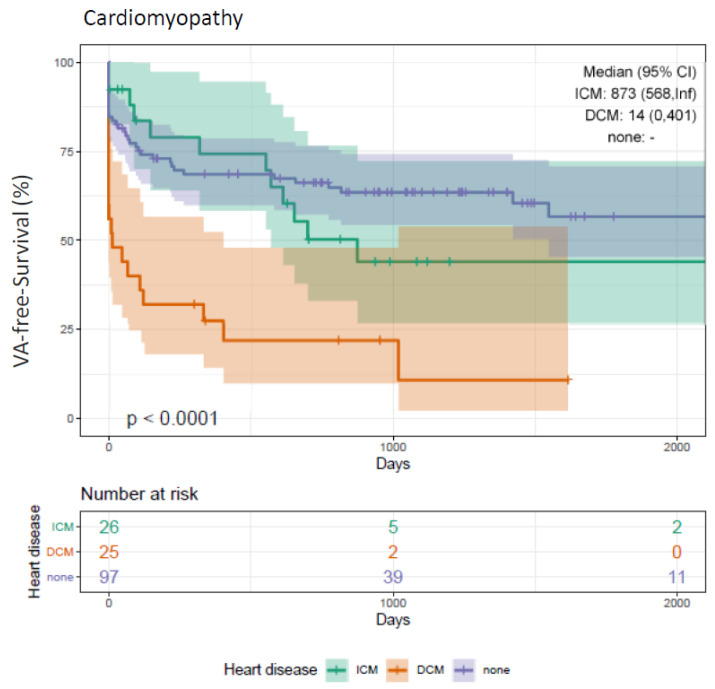
Kaplan–Meier plot on freedom from VA recurrence in patients undergoing RMN ablation with cardiomyopathy (DCM, ICM) compared to patients without. Patients undergoing RMN ablation for VA with DCM present with a significantly worse outcome compared to patients with ICM and patients without cardiomyopathies (DCM, ICM) (log-rank *p*-value < 0.001*). VA, ventricular arrhythmia; VT, ventricular tachycardia; PVC, premature ventricular contractions; RMN, remote magnetic navigation; ICM, ischemic cardiomyopathy; DCM, dilated cardiomyopathy; None, absence of cardiomyopathy (DCM, ICM); FU, follow-up.

Concerning VA-free survival, significant differences were observed depending on the diagnosis of cardiomyopathy. DCM patients presented with the worst outcome in terms of a significantly higher risk for VA recurrence (Log-rank *p* < 0.001*) (Figure 3). Compared to patients with ICM, VA-free survival was better in patients with idiopathic VA without reaching statistical significance (*p* = 0.240).

Uni- and multivariate Cox regression analyses found DCM to be an IP for VA recurrence associated with a 3.7-fold increase in recurrent VA. ICM patients presented with a 56% increase in VA recurrence (Table 2 and Table 3).

Baseline AAD class I/III/IV intake was identified as IP for VA recurrence (Table 2 and Table 3), too.

### 3.11. Clinical Outcome Depending on Beta Blocker and AADs Class I/III/IV

Far more patients with VT received AADs class I/III/IV compared to PVC patients at baseline (Supplementary Table S1).

Compared to patients without cardiomyopathies (DCM, ICM), beta blocker and AADs class I/III/IV were both far more often prescribed in patients with cardiomyopathies (Supplementary Table S2).

**Figure 4:**
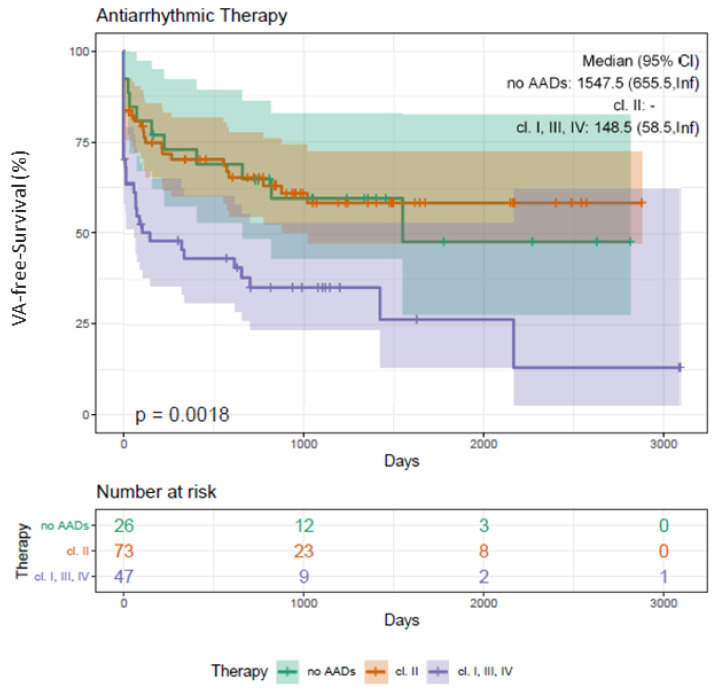
Kaplan–Meier plot on freedom from VA recurrence in patients undergoing RMN ablation depending on the administration of AADs class I/III/IV and beta blocker. Patients undergoing RMN ablation for VA with AAD class I/III/IV intake present with a significantly worse outcome compared to patients with beta-blockers or without medical antiarrhythmic therapy (log-rank *p*-value = 0.002*). VA, ventricular arrhythmia; VT, ventricular tachycardia; PVC, premature ventricular contractions; RMN, remote magnetic navigation; AADs, antiarrhythmic agents; FU, follow-up.

Significant differences were observed in the latter (ICM: *n* = 24, 77%; DCM: *n* = 11, 42%; *p* = 0.015*) at baseline (Supplementary Table S3). Patients without previous AAD class I/III/IV treatment had an improved outcome compared to patients with AAD class I/III/IV prescription (log-rank *p* = 0.002*; Figure 4). Uni- and multivariate analyses confirmed previous AAD class I/III/IV therapy as IP for VA recurrence (*p* = 0.030*, HR 2.48, CI 1.11–5.68; Table 2 and Table 3).

### 3.12. Clinical Outcome and Sex Disparities

**Figure 5:**
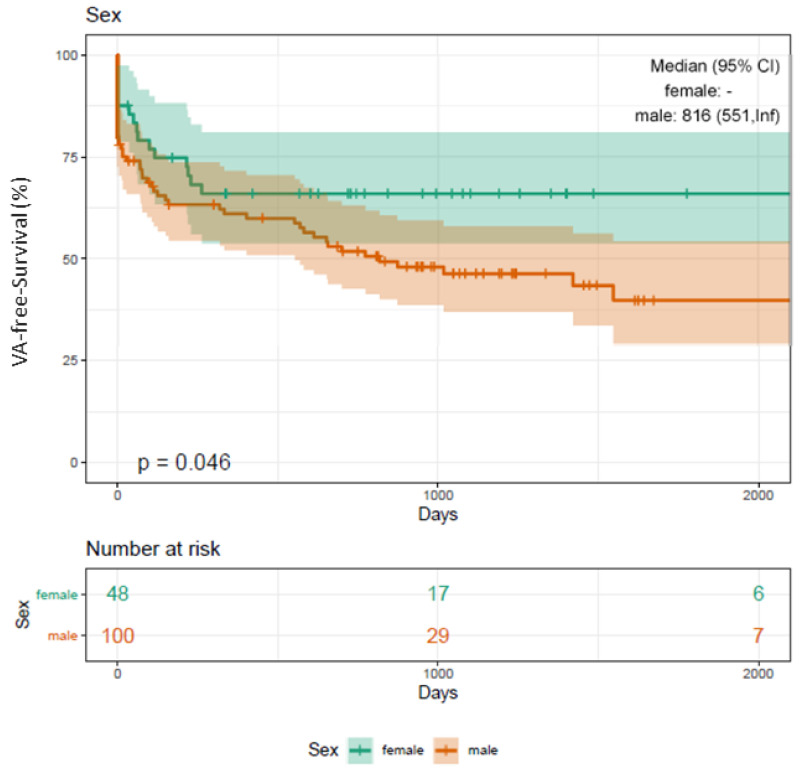
Kaplan–Meier plot on freedom from VA recurrence in patients undergoing RMN ablation depending on sex. Male patients undergoing RMN ablation for VA present with a significantly worse outcome compared to female patients (Log-rank *p*-value = 0.046*). VA, ventricular arrhythmia; VT, ventricular tachycardia; PVC, premature ventricular contractions; RMN, remote magnetic navigation; FU, follow-up.

Significantly more male patients (*n* = 40, 93%) suffered from sustained VT (female *n* = 3, 7%; *p* < 0.001*; Supplementary Table S1) and presented with cardiomyopathies (DCM, ICM) (male *n* = 58, 86%; female *n* = 9, 13%; *p* < 0.001*; Supplementary Table S2). Sex disparities are also depicted in Figure 5.

### 3.13. Complications

Major complications requiring intervention occurred in 15 patients (9%). These complications consisted of four patients with intraprocedural cardiopulmonary resuscitation (2%) due to ventricular fibrillation, one patient with cardiogenic shock (<1%), six patients with pericardial effusion (3%) with three of these patients developing a pericardial tamponade (2%), two patients with a steam-pop phenomenon (<1%), one patient with a new right bundle bunch block (<1%) and one patient with a third-degree AV-block (<1%). All of these complications were treated successfully. None of the patients died in the course of procedure-related severe adverse events.

## 4. Discussion

### 4.1. Main Findings

This study aimed to evaluate long-term freedom from VA recurrence after RMN-guided ablation and to identify risk factors for VA recurrence in a preselected cohort of patients.

Our study has four major findings. First, RMN ablation of VT and PVC patients can be performed with comparable safety profile and effectiveness as conventional procedures. Second, HFrEF patients presented with a significantly worse outcome compared to patients with an EF > 40%. Third, recurrence rates in DCM patients were significantly higher following RMN-guided ablation in contrast to ICM patients. Fourth, DCM and baseline AAD class I/III/IV therapy were identified as IPs for VA recurrence using RMN.

### 4.2. VA-Free Survival Using RMN

The procedural success rates (defined as non-inducibility of any VA) using RMN ranges from 52% to 86%, with VT recurrence rates between 14% and 50% during a median follow-up of 9 to 22 months [3,4,5,6,7,8]. We report on a quite similar procedural success rate of 82% (144 patients) and an overall recurrence rate of 39% (69 patients). In contrast to the studies mentioned above, we analyzed long-term outcome data for an extended period of up to 66 months (Figure 1, Figure 2, Figure 3, Figure 4 and Figure 5).

Our acute procedural success rate of 82% did not differ significantly in patients suffering from cardiomyopathies (DCM, ICM) (83%, 56 patients) and idiopathic VT (81%, 86 patients). Significant differences between these groups first became obvious during follow-up (Figure 3). This could be explained by the underlying individual arrhythmia substrate and pattern in patients with cardiomyopathy and the natural course of the disease. In this context, the authors of the HELP-VT study emphasize the importance of intraprocedural complete identification and elimination of arrhythmia substrates to improve patients’ outcomes [6].

Recurrence rates from manual VT ablations with a 1-year recurrence rate of 53% are described as reasonably comparable to recurrence rates from RMN-guided ablation between 14% and 50%, with a trend toward lower recurrence rates after RMN ablation [4,5,6,7,8,9,10]. A large meta-analysis reports on improved results following RMN compared to manual guided navigation in terms of fluoroscopy and procedure time, acute procedural success, complications, and long-term VT recurrence [11].

In the meta-analysis mentioned above and in another study, RMN significantly reduced mean fluoroscopy (mean difference: −10 min) as well as mean procedure time (mean difference: −10 min) [11,12].

This is in line with our data reporting a mean fluoroscopy time of 5 min, which resulted in a relevant reduction in radiation exposure for the patient and operator.

Although VA ablation procedures are often complex requiring individual ablation approaches, the mean procedure duration in our study was 206 ± 88 min and thus even similar to procedure times reported from RMN-guided AF ablation procedures [12,13,14], reflecting the benefit of RMN taking into account a certain learning curve and experience of the operator.

Using manual techniques in experienced centers, major complications were reported in 4% to 15% of cases, including in-hospital death in 4.8% [6,10,15,16]. In some studies, the risk of major complications has been shown to be lower in RMN -guided procedures relative to manual technique [12,14]. We present a major complication rate of 8% without any in-hospital deaths. Thus, our data act in concert with these observations.

In addition, no disturbances of ICD/CRT-D functions in relationship to RMN were observed. These findings are in line with results from previous studies, excluding significant effects on device system integrity when using RMN [17,18].

### 4.3. VA-Free Survival (PVC vs. VT)

Patients undergoing ablation for recurrent PVC presented with improved VA-free survival compared to patients suffering from recurrent VT (Figure 1), reflecting the serious morbidity and the increased risk of mortality of VT patients compared to PVC patients.

### 4.4. VA-Free Survival (DCM vs. ICM)

Although acute procedural outcome has been reported to be similar for ischemic and non-ischemic etiologies [5], VT recurrence rates are usually higher in DCM. The HELP-VT study described one-year freedom from VT recurrence rate of 40.5% in DCM compared to 57% in ICM [6].

We observed differences between ICM and DCM, too. Figure 3 demonstrates an initially very steep slope in DCM patients, which represents an increased rate of early recurrences (Figure 3). In line with these results, DCM patients presented with a significantly lower reduction in their PVC burden (DCM: −37%, *p* = 0.008; ICM −68%, *p* = 0.002*).

In summary of these findings, our data clearly show that the management of VT in non-ischemic cardiomyopathies remains challenging due to an ongoing underlying disease and atypical scar formation [6,19,20]. Pre-ablation LGE CMR imaging might help to improve procedural success [21] and to reduce the risk for ICD shock, which is associated with a significantly increased risk of death [22,23].

### 4.5. VA-Free Survival (Idiopathic VT)

Single-center studies have reported favorable procedural success rates for idiopathic VT ablation ranging from 80% to 93% [24,25,26,27,28,29]. These findings are in line with our results, as demonstrated in Figure 3. Significant differences are largely attributed to the underlying VT mechanism. In contrast to reentry with multiple exit sites in scar-related VT, it is a focal triggered activity in idiopathic VT.

As in structurally normal hearts, the reported outcomes of catheter ablation are very suitable. These patients should be referred earlier for ablation, which can be considered as first-line therapy in case of arrhythmia-related symptoms [3].

### 4.6. Additional Predictors of VA Recurrence

In our study, far more patients with VT (31 cases, 72%) received AADs class I/III/IV compared to PVC patients (24 cases, 18%) (*p* < 0.001*), most likely reflecting progressed heart disease with greater arrhythmia substrates and more frequent malignant arrhythmias in VT patients.

Referring to AADs class I/III/IV in idiopathic VT, VA success rates of 71% off medication and of 85% with antiarrhythmic drugs over a mean follow-up of 1.9 years were reported [28].

In our study, AADs class I/III/IV were far more often prescribed in patients with cardiomyopathies. We documented a significantly lower VA-free survival rate in patients with baseline AAD class I/III/IV administration (log-rank *p* = 0.002*) (see Figure 4) and identified baseline AAD class I/III/IV therapy as IP for VA recurrence (Table 2 and Table 3). One reason for these results might be an advanced state of cardiomyopathies (DCM, ICM) in those patients prescribed with AADs class I/III/IV as well as the reported limited success rate of an AAD class I/III/IV treatment in the setting of recurrent symptomatic VT [20].

Referring to the results of the VANISH trial on patients’ outcome catheter ablations for VT clearly outclass success rates of AAD class I/III/IV therapies in ICM patients [30].

Concerning ablation approaches, RMN has become a widely accepted means of treating a variety of arrhythmias. Numerous publications demonstrated that the technology may be associated with significant safety benefits for the patient and the physician [13]. Nevertheless, there is still the need for further improved treatment strategies, as confirmed by our study.

Beneficial therapeutic effects of RMN ablation and AADs class I/III/IV are limited. Recurrent ICD therapy is associated with an increased risk of mortality and impaired myocardial function [22,23], and CRT-D therapy only appears to reduce new onset VT [31]. Thus, further optimized treatment strategies are required.

### 4.7. Clinical Perspective and Translational Outlook

Because of the impact of recurrent VA on cardiovascular morbidity and mortality, further studies focusing on modifications of treatment strategies are required. The influence of specific biomarkers associated with the development of fibrosis has to be analyzed, and these effects need to be translated into clinics. Future studies have to focus on different etiologies of DCM as they have important practical and prognostic implications VA.

## 5. Conclusions

VT, an impaired LVEF (%), the need for AAD class I/III/IV therapy, as well as cardiomyopathies, has relevant effects on arrhythmia recurrence after RMN-guided ablation. Our data demonstrate an improved long-term outcome in idiopathic VT patients compared to patients with cardiomyopathies (DCM, ICM). DCM patients were found to be at high risk for VA recurrence due to scar location and distribution. Further personalized paths in arrhythmia management are needed in this specific cohort of patients.

## Figures and Tables

**Table 1:** Baseline characteristics.

Characteristics	*n* = 176
Age (years)	53.23 ± 17.55
Sex, female	65 (37%)
BMI (kg/m^2^)	26.71 ± 4.25
LVEF (%)	48.22 ± 10.8
Cardiomyopathy	57 (32%)
ICM	31 (18%)
DCM	26 (15%)
Valve disease	43 (24%)
Hypertension	98 (56%)
Diabetes mellitus	22 (13%)
Beta blocker BL	125 (71%)
AADs class I/III/IV BL	56 (32%)
ICD	24 (14%)
CRT-D	24 (14%)
Renal failure	23 (13%)
History of stroke	11 (6%)

Continuous variables are shown as the mean ± SD and categorical variables as the number (%). BMI, body mass index; LVEF, left ventricular ejection fraction; ICM, ischemic cardiomyopathy; DCM, dilated cardiomyopathy; BL, baseline; AADs, antiarrhythmic agents; ICD, implantable cardioverter defibrillator; CRT-D; cardiac resynchronization therapy defibrillator.

**Table 2:** Univariate Cox regression analysis of risk factors for VA recurrence of patients undergoing RMN ablation.

Risk Factor	Hazard Ratio	*p*-Value
ICM	1.49 (0.79–2.81)	0.221
DCM	3.80 (2.18–6.64)	>0.001 *
LVEF < 40%	2.06 (1.26–3.35)	0.004 *
AADs class I/III/IV BL	2.27 (1.14–4.52)	0.020 *
Beta blocker BL	0.97 (0.48–1.96)	0.942
Sex (female)	0.58 (0.33–1.01)	0.055
VT ablation	1.9 (1.15–3.12)	0.012 *
GFR <60 mg/dl/min	1.45 (0.8–2.62)	0.221

VA, ventricular arrhythmias; ICM, ischemic cardiomyopathy; DCM, dilated cardiomyopathy; LVEF, left ventricular ejection fraction; AADs, antiarrhythmic agents; BL, baseline; VT, ventricular tachycardia. * and bold letters indicate statistical significance.

**Table 3:** Multivariate Cox regression analysis of risk factors for VA recurrence of patients undergoing RMN ablation.

Risk Factor	Hazard Ratio	95% Lower	95% Upper	*p*-Value
LVEF < 40%	1.11	0.45	2.76	0.821
DCM	3.74	1.58	8.88	<0.01 *
AADs class I/III/IV BL	2.48	1.11	5.68	0.03 *
VT ablation	1.02	0.53	1.99	0.94

VA, ventricular arrhythmias; LVEF, left ventricular ejection fraction; DCM, dilated cardiomyopathy; ICM, ischemic cardiomyopathy; AADs, antiarrhythmic agents; BL, baseline; VT, ventricular tachycardia. * and bold letters indicate statistical significance.

## Data Availability

The data underlying this article will be shared upon reasonable request to the corresponding author.

## Supplementary Materials

The following are available online at https://www.mdpi.com/article/10.3390/jcm10204695/s1, Table S1: Baseline characteristics of patients undergoing VA ablation (PVC compared to VT), Table S2: Baseline characteristics of VA patients with and without cardiomyopathies (DCM, ICM), Table S3: Baseline characteristics of VA patients with cardiomyopathy (DCM compared to ICM), Table S4: Baseline symptoms of VA patients.
