# Supplementary material for: Long-Term Efficacy and Impact on Mortality of Remote Magnetic Navigation Guided Catheter Ablation of Ventricular Arrhythmias
Source: J Clin Med. 2021 Oct 13;10(20):4695. doi: 10.3390/jcm10204695 (PMC8540658; doi:10.3390/jcm10204695)
Supplement: Supplementary file 1 [file jcm-10-04695-s001.zip › jcm-1386565-supplementary.pdf]

Supplementary Table 1. Baseline characteristics of patients undergoing VA ablation (PVC compared to VT).

Characteristics	PVC (*n* = 132)	VT (*n* = 44)	*p*-Value
Age (years)	53.30 ± 15.97	52.49 ± 21.81	0.52
Sex, female	62 (47%)	3 (7%)	**<0.01 ***
BMI (kg/m^2)	27.07 ± 4.53	25.64 ± 3.05	**0.02 ***
LVEF (%)	50.65 ± 8.28	40.64 ± 11.84	**<0.01 ***
Cardiomyopathy	28 (21%)	29 (66%)	**<0.01 ***
ICM	10 (8%)	20 (46%)	**<0.01 ***
DCM	18 (14%)	8 (18%)	0.08
Valve disease	27 (20%)	16 (36%)	**<0.01 ***
Hypertension	70 (53%)	27 (62%)	**0.04 ***
Diabetes mellitus	16 (12%)	6 (13%)	**0.01 ***
Beta blocker BL	93 (70%)	31 (71%)	0.97
AADs class I/III/IV BL	24 (18%)	31 (71%)	**<0.01 ***
ICD	10 (8%)	14 (32%)	**<0.01 ***
CRT-D	9 (7%)	15 (34%)	**<0.01 ***
Renal failure	10 (8%)	12 (27%)	**<0.01 ***
History of stroke	6 (12%)	5 (11%)	0.06

Continuous variables are shown as the mean ± SD and categorical variables as the number (%). VA, ventricular arrhythmias; PVC, premature ventricular contractions; VT, ventricular tachycardia; BMI, body mass index; LVEF, left ventricular ejection fraction; ICM, ischemic cardiomyopathy; DCM, dilated cardiomyopathy; BL, baseline; AADs, antiarrhythmic agents; ICD, implantable cardioverter defibrillator; CRT-D; cardiac resynchronization therapy defibrillator. *and bold letters indicate statistical significance.

Supplementary Table 2. Baseline characteristics of VA patients with and without cardiomyopathies (DCM, ICM).

Characteristics	None (*n* = 119)	Cardiomyopathy (*n* = 57)	*p*-Value
Age (years)	47.02 ± 18.19	63.33 ± 10.40	**<0.01 ***
Sex, female	56 (47%)	9 (16%)	**<0.01 ***
BMI (kg/m^2)	26.24 ± 4.55	27.47 ± 3.60	**0.02 ***
LVEF (%)	53.50 ± 4.06	39.62 ± 11.25	**<0.01 ***
Cardiomyopathy	0 (0%)	57 (100%)	**<0.01 ***
ICM	0 (0%)	31 (54%)	**<0.01 ***
DCM	0 (0%)	26 (46%)	**<0.01 ***
Valve disease	17 (14%)	26 (46%)	**<0.01 ***
Hypertension	41 (35%)	57 (100%)	**<0.01 ***
Diabetes mellitus	8 (7%)	14 (25%)	**0.02 ***
Beta blocker BL	65 (55%)	57 (100%)	**<0.01 ***
AADs class I/III/IV BL	20 (17%)	36 (63%)	**<0.01 ***
ICD	7 (6%)	17 (30%)	**<0.01 ***
CRT-D	0 (0%)	24 (42%)	**<0.01 ***
Renal failure	3 (3%)	8 (14%)	**0.02 ***
History of stroke	3 (3%)	14 (25%)	**0.03 ***

Continuous variables are shown as the mean ± SD and categorical variables as the number (%). VA, ventricular arrhythmias; BMI, body mass index; LVEF, left ventricular ejection fraction; ICM, ischemic cardiomyopathy; DCM, dilated cardiomyopathy; BL, baseline; AADs, antiarrhythmic agents; ICD,

implantable cardioverter defibrillator; CRT-D; cardiac resynchronization therapy defibrillator. *and bold letters indicate statistical significance.

Supplementary Table 3. Baseline characteristics of VA patients with cardiomyopathy (DCM compared to ICM).

**Characteristics**	**DCM (*n* = 26)**	**ICM (*n* = 31)**	***p*-Value**
Age (years)	62.54 ± 8.70	65.39 ± 9.00	0.34
Sex, female	4 (15%)	1 (3%)	0.20
BMI (kg/m^2)	27.57 ± 3.67	27.09 ± 3.02	0.59
LVEF (%)	39.52 ± 10.68	35.95 ± 10.67	0.15
Valve disease	11 (42%)	15 (48%)	0.28
Hypertension	20 (77%)	28 (90%)	0.31
Diabetes mellitus	7 (27%)	7 (23%)	0.94
Beta blocker BL	23 (88%)	27 (87%)	1.00
AADs class I/III/IV BL	11 (42%)	24 (77%)	**0.01** *
ICD	5 (19%)	11 (35%)	0.28
CRT-D	15 (58%)	9 (29%)	**0.05** *
Renal failure	6 (23%)	14 (45%)	0.14
History of stroke	1 (4%)	7 (23%)	0.09

Continuous variables are shown as the mean ± SD and categorical variables as the number (%).VA, ventricular arrhythmias; ICM, ischemic cardiomyopathy; DCM, dilated cardiomyopathy; BMI, body mass index; LVEF, left ventricular ejection fraction; BL, baseline; AADs, antiarrhythmic agents; ICD, implantable cardioverter defibrillator; CRT-D; cardiac resynchronization therapy defibrillator. *and bold letters indicate statistical significance.

Supplementary Table 4. Baseline symptoms of VA patients.

**Symptoms**	**(*n* = 176)**
Palpitations	126 (72%)
Dizziness	66 (38%)
Dyspnea	69 (39%)
Syncope	19 (11%)

Categorical variables are shown as the number (%).VA, ventricular arrhythmias.
